# *Sell*^*hi*^ neutrophils: a key to triumph in cancer therapy

**DOI:** 10.1038/s41392-023-01671-6

**Published:** 2023-11-08

**Authors:** Ekaterina Pylaeva, Jadwiga Jablonska

**Affiliations:** 1 Department of Otorhinolaryngology, University Hospital Essen, University Duisburg-Essen, Essen, 45147 Germany; 2 German Cancer Consortium (DKTK) partner site Düsseldorf/Essen, Essen, 45147 Germany

**Keywords:** Tumour immunology, Innate immunity

In a recent study published in *Cell*, Gungabeesoon and colleagues^1^ demonstrated that a specific subpopulation of neutrophils, characterized by a distinct *Sell*^*hi*^ (CD62L^hi^) phenotype with an interferon gene signature, acutely accumulates in tumors during successful immunotherapy and is associated with better treatment outcome. These findings enhance our understanding of the neutrophil heterogeneity within the tumor context and highlight their potential in cancer immunotherapy.

Traditionally, neutrophil infiltration of tumor tissue has been primarily associated with tumor progression. However, the remarkable heterogeneity of neutrophils allows them to play multiple roles in this process. In addition to their typical pro-angiogenic and immunosuppressive functions, neutrophils can also have protective roles, such as direct cancer cell killing or stimulation of adaptive immune responses. Importantly, various anti-cancer therapies directly or indirectly (by modulation of tumor microenvironment), impact neutrophil responses and activity. Nevertheless, some immunotherapies fail to induce clinical responses in certain patients, prompting extensive efforts to understand the underlying mechanisms.

**Fig. 1:**
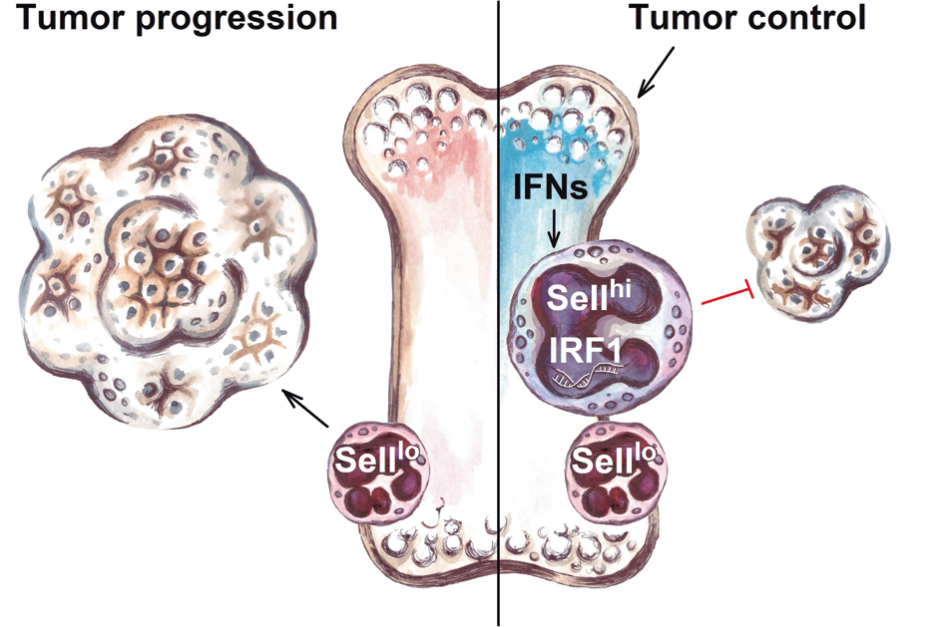
Successful tumor control after immunotherapy is associated with the accumulation of *Sell*^*hi*^ neutrophils characterized with interferon-stimulated gene signature and anti-tumor properties. IFNs interferons, IRF1 interferon regulatory factor 1

To address this, Gungabeesoon et al.^1^ analyzed neutrophil phenotypes expanded after successful immunotherapy. These cells had been previously overlooked in single-cell transcriptomic research due to unintended elimination during standard preparation of the sample. The authors observed that therapy-induced neutrophils expressed high levels of *Sell* transcripts, encoding the protein Selectin L (also known as CD62L). These neutrophils exhibited a more immature phenotype (CD101^lo^), associated with increased cytotoxicity and upregulated expression of genes responsible for regulation of neutrophil degranulation, suggesting their potential immunostimulatory activity. This aligns with prior observations indicating anti-tumoral properties of immature neutrophil populations.^2^ *Sell*^*hi*^ neutrophils also exhibited a distinct interferon (IFN) signature, including interferon regulatory factor 1 (IRF1), a crucial driver of their anti-tumor phenotype, and appeared to be essential for the successful therapy (Fig. 1).

IFN signatures have previously been shown to govern neutrophils anti-tumoral activity, such as cytotoxicity and immunostimulatory properties. IFN signaling has been shown to be essential for the effect of β-glucan-induced trained immunity, associated with neutrophil reprogramming towards the immunostimulatory phenotype.^3^ In agreement, disturbed IFN signaling has been linked to pro-tumoral activation of neutrophils and accelerated tumor progression in IFN-deficient hosts. Furthermore, the cancer environment seems to actively contribute to the downregulation of IFN signaling through the degradation of the IFN receptor.

The findings by Gungabeesoon et al.^1^ contribute to our understanding of neutrophil polarization in cancer and their heterogeneity. While the concept of a binary anti-tumoral/ pro-tumoral neutrophil polarization oversimplifies the continuum of neutrophil phenotypes in cancer, it surely allows easier understanding of the complex plasticity of these cells. Neutrophils are adaptable cells gaining various properties depending on the microenvironmental cues. Emerging data indicate that either anti- or pro-tumor neutrophils coexist in tumor situation, and the balance between these populations, rather than a single population, contributes to disease progression. Importantly, factors such as IFNs drive the shift along this continuum.

Another significant aspect of this study concerns the acute expansion of tumor neutrophil numbers after successful therapies. It was previously postulated that neutrophil depletion or the blockade of their tumor infiltration should be achieved in order to eliminate tumors. However, recent studies indicate the anti-tumoral, immunostimulatory role of neutrophils infiltrating tumor^2^ or tumor-draining lymph nodes^4^ during the early stages of disease, contributing to improved patient outcomes. Gungabeesoon et al.^1^ suggest that pro- and anti-tumor neutrophils do not differentiate from each other but rather have distinct origins shaped by microenvironmental factors, including activated dendritic cells, IFNg and IL12. The existence of diverse neutrophil phenotypes in tumor tissue implies that therapeutic re-polarization of neutrophils, rather than their depletion, should be pursued to achieve efficient therapy outcomes. Broad neutrophil depletion strategies carry the risk of eliminating both beneficial and detrimental cells. Therefore, a deeper understanding of the regulatory mechanisms responsible for acquiring distinct neutrophil states is needed to allow selective manipulation of neutrophil subpopulations. The data from Gungabeesoon et al.^1^ shed new light on the regulation of neutrophil polarization during therapy and likewise advocate for neutrophil repolarization approaches to enhance therapeutic efficacy.

The findings of this manuscript also raise intriguing questions regarding when and where neutrophil education occurs. The increased abundance of the *Sell*^*hi*^ neutrophils in the bone marrow and their presence in bloodstream suggest that neutrophil education begin during neutrophil development in the bone marrow microenvironment. Such bone marrow neutrophil training has been previously suggested.^2^ The possibility of bone marrow-based neutrophil education raises further questions about the role of the cancer microenvironment in regulating the activity of these cells.

The authors also noted that another population of neutrophils, *Sell*^*lo*^ (*Siglecf*^*hi*^), was sustained in tumors after treatment, even as *Sell*^*hi*^ neutrophils increased. SiglecF^hi^ neutrophils were previously shown to have pro-tumorigenic properties.^5^ Here the authors showed that these neutrophils were characterized by multiple gene signatures that support their pro-tumorigenic roles, including expression of genes that promote tumor proliferation, remodeling of extracellular matrix, angiogenesis, suppression of antitumor immunity, and recruitment of myeloid cells, and thus reported to be responsible for metastasizing, sustained in tumors after treatment. Therefore, this study suggests that therapies should aim not only to expand anti-tumor neutrophils, but also to eliminate pro-tumor counterparts. Further human studies will be needed however to understand the dynamics of these populations in a relevant clinical setting following therapy. Human studies should also allow evaluating whether the abundance of these neutrophil populations predict progression and outcome in human cancers.

Single-cell transcriptomics has revolutionized our understanding of neutrophil biology by unraveling the phenotypical and functional diversity of neutrophil subsets across various organs. The pursuit of selective strategies to therapeutically enhance beneficial neutrophil populations while concurrently eliminating tumor-supporting neutrophils remains a compelling approach in immunotherapy. Rather than complete depletion of neutrophils, successful immunotherapy should focus on modulating the tumor microenvironment to favor anti-cancer repolarization of neutrophils. This approach holds the potential to establish an effective strategy for achieving optimal anti-tumor responses and enhancing the outcomes of cancer patients.
